# Self-medication in Chinese residents and the related factors of whether or not they would take suggestions from medical staff as an important consideration during self-medication

**DOI:** 10.3389/fpubh.2022.1074559

**Published:** 2022-12-22

**Authors:** Pu Ge, Qiyu Li, Murong Dong, Yuyao Niu, Xiao Han, Ping Xiong, Yuhan Bao, Hewei Min, Diyue Liu, Suqi Wang, Jinzi Zhang, Ziwei Zhang, Wenli Yu, Xinying Sun, Lian Yu, Yibo Wu

**Affiliations:** ^1^Institute of Chinese Medical Sciences, University of Macau, Taipa, Macao SAR, China; ^2^School of Humanities and Health Management, Jinzhou Medical University, Jinzhou, China; ^3^Faculty of Education, University of Malaya, Kuala Lumpur, Malaysia; ^4^Faculty of Arts and Humanities, University of Macau, Taipa, Macao SAR, China; ^5^Department of Pharmacy, The Fifth Affiliated Hospital of Sun Yat-sat University, Zhuhai, China; ^6^Health Clinic, Changzhou Institute of Technology, Changzhou, China; ^7^School of Public Health, Peking University, Beijing, China; ^8^International School of Public Health and One Health, Hainan Medical University, Haikou, China; ^9^School of Philosophy, Anhui University, Hefei, China; ^10^School of Humanities and Social Sciences, Harbin Medical University, Harbin, China; ^11^School of Foreign Languages, Weifang University of Science and Technology, Weifang, China; ^12^School of Public Health, Xi'an Jiaotong University, Xi'an, China

**Keywords:** self-medication, over-the-counter drugs (OTC), medical staff advice, Big Five personality, health literacy, self-efficacy, health condition, medical staff

## Abstract

**Objective:**

To investigate the status of Chinese residents' self-medication behavior and the important factors to consider when purchasing OTC drugs, and to explore the related factors of the possibility that Chinese residents take medical staff's suggestions as important factors to consider when purchasing OTC drugs.

**Study design:**

A cross-sectional survey.

**Methods:**

A questionnaire was developed for exploring the sociodemographic characteristics of the respondents, their self-medication status, and important considerations. The questionnaire includes several scales including Health Literacy Scale-Short Form (HLS-SF), EQ-5D Visual Analog Scale (EQ-5D-VAS), Big Five Inventary-10 Items (BFI-10), and New General Self Efficacy Scale (NGSES). After carrying out a multi-stage sampling method, the questionnaire was conducted nationwide from July 10 to September 15, 2021. Next, descriptive statistics were conducted to analyze the general features. Logistic regression was then used to analyze the related factors of the possibility that the respondents took the suggestions of medical staff as an important consideration when purchasing OTC drugs.

**Results:**

Nine thousand two hundred fifty-six qualified questionnaires were received. 99.06% of Chinese adults had self-medication behaviors. The types of OTC drugs purchased most by the respondents were NSAIDs (5,421/9,256 people, 58.57%) and vitamins/minerals (4,851/9,256 people, 52.41%). 86.2% of the respondents took the suggestions of medical staff as an important consideration when purchasing OTC drugs. The results of multi-factor logistic regression showed that women, those living in the central and western regions of China, those suffering from chronic diseases, those with high agreeableness, high conscientiousness, high neuroticism and openness, high health literacy, high EQ-5D-VAS, and those with high self-efficacy are more likely to take medical staff's suggestions as important factors to consider.

**Conclusion:**

The vast majority of Chinese adults have self-medication behavior. Important considerations when purchasing OTC drugs include medical staff's suggestions, drug safety and drug efficacy. Whether residents take the suggestions of medical staff as an important consideration is related to their sociological characteristics, agreeableness, conscientiousness, neuroticism, openness, health literacy, self-assessment health status, and self-efficacy. When purchasing and using OTC drugs, residents should carefully listen to the suggestions from medical staff. They should also carefully consider their own conditions before buying OTC drugs.

## 1. Background

Self-medication is defined by The World Health Organization (WHO) as an individual behavior in which people use drugs to treat their self-recognized diseases or symptoms. Reasonable self-medication is beneficial for individuals in terms of disease prevention and disease treatment (1). Residents are typically discovered to self-medicate with over-the-counter drugs (OTC) which refer to safe and effective drugs that can be purchased without a prescription and can be taken by consumers themselves according to the instructions (2). Beneficially, the use of OTC drugs reduces unnecessary or inappropriate hospital visits of residents (3), which, to a certain extent, reduces residents' demand for hospital visits and expands residents' right to choose treatment options independently. Since 2019, due to the decreasing number of residents visiting hospitals caused by the outbreak of the Coronavirus disease 2019 (i.e., COVID-19) pandemic and the relative availability of OTC drugs online or offline, there was an increase in the rate of self-medication among Chinese residents (4).

Globally, the authorities of different countries have taken diverse measures to regulate citizens' behavior of self-medication. Specifically, in 2017, the Japanese government introduced a policy on OCT drug use, demonstrating that if a resident purchases a “switched OTC drug” (i.e., an OTC drug converted from a prescription drug), the payment for the drug can be reduced or exempted under the self-use levy system (5). In America, the US Food and Drug Administration (FDA) has established an expanded task force on the regulation of OTC safe use and a national regulatory agency website to ensure the quality of OTC medicines (3). Also, the FDA required all OTC products should include details such as the indications of the drug, the contraindications of the drug, and the precautions when using the drug over their outer packaging (6). In 2000 in China, the “Administrative Measures for the Classification of Prescription Drugs and Non-prescription Drugs” issued by the former China Food and Drug Administration (CFDA) regulated China's pharmaceuticals industry in terms of how to allocate, purchase and use prescription drugs as well as on how to label, instruct, package, print and sell non-prescription drugs (7). Later in 2004, the CFDA issued the “Notice on Carrying out the Evaluation of the Conversion of Prescription Drugs and Non-Prescription Drugs” to implement adynamic management of the non-prescription drug list (8). Most recently in 2021, the China OTC Drug Association proposed the development plan for OTC drug industry of China; the proposal clarified the medical, social, and economic value of the OTC drugs, and pointed out the problems existing in the management of non-prescription drugs in China (9). In light of the foregoing, it can be seen that various countries, including China, have adopted relevant policies to regulate residents' self-medication behavior and improve the quality and accessibility of OTC drugs.

Self-medication is becoming an important part of residents' health care (10). The advantages of rational self-medication would be time-and-cost-saving, timely treatment of various diseases, and even saving lives (11). However, incorrect self-medication can be harmful to an individual's health (10). For example, drug abuse in residents' self-medication can further lead to drug addiction and dependence (12). According to a national survey in the US in 2008, the overuse of OTC cold-cough drugs, medicines containing morphine, codeine, papaverine, thebaine, noscapine, and other alkaloids, occurred to around one million residents (5.3%) in a year (13).

The term medical staff refers to a person who has received formal medical training and is experienced to provide treatment and advice related to health care; those health professionals mainly include doctors, pharmacists, nurses, medical technicians, and so on (14–16). Among them, doctors and pharmacists are the main providers of drug related knowledge for patients. In China, physicians have the right to prescribe drugs while pharmacists take charge of the review of prescriptions, and the deployment and verification of drugs (17). The false advertisements on radio, television, Internet, and other media, as well as suggestions from relatives and friends who are non-medical staff, are considered to be unreliable sources of information for purchasing OTC drugs. If residents are induced to buy and use OTC drugs by these unreliable sources of information, they may face the risks of delay in regular treatment, which may further aggravate their conditions. A number of studies have revealed a high proportion of residents following medical staff advice when purchasing OTC drugs, and it is highlighted that the medical staff's advice is one of the most critical factors that residents need to consider when seeking drug treatment (18).

Based on the above statements, one aim of the current study is to explore the related factors of the possibility that residents take medical staff's advice as an important factor to consider when purchasing OCT drugs. The investigation involved several important independent variables including self-efficacy, health literacy, health status, and personality traits, as well as the demographic features of the population included in the study. Self-efficacy refers to the degree of individual's self-confidence in their ability to complete tasks and achieve goals (19); health literacy, refers to individuals' ability to acquire and understand information related to health and the ability to use this information to maintain and promote their health (20); the participants' health status was measured by the health effect value visual analog scale (VAS) which is part of the European five-dimensional health scale (EQ-5D-5L); this scale would be used to assess the health status of residents (21). The personal traits would be measured based on the Big Five personality model or The Five Factor Model (FFM), which is a taxonomy or a grouping of personality traits that include openness, agreeableness, neuroticism, conscientiousness, and extroversion (22).

Additionally, the research seeks to investigate the current status of Chinese residents' self-medication behavior, including the rate of residents' self-medication and the types of drugs they purchased and used by themselves. Particularly, this research concentrates on the related factors of the possibility of taking medical staff's advice as an important consideration when purchasing OTC drugs, to provide a reference for Chinese government when issuing policies to regulate the self-medication behavior of residents and to promote the rational self-medication behaviors.

## 2. Methods

### 2.1. Study participants

The research data comes from the 2021 China Family Health Index Survey (China Family Health Index-2021, CFHI-2021) (23). The survey is based on multi-stage sampling across the country. When selecting cities, all the provincial capital cities of provinces and autonomous regions, as well as municipalities in China were firstly included. Later, the random-number table was applied to randomly select the non-provincial capital cities of all provinces and autonomous regions in the country. Finally, 120 cities were selected within China. During the second phase of sampling, the population of each city was stratified according to gender, age, and urban-rural distribution, and the sample size of each stratum was 100 people, which was determined according to the demographic characteristics of the “Seventh National Census in 2021” (24). Convenience sampling was carried out on the premise of meeting quota requirements. After the completion of the sampling, with the favor of the investigator recruited in each city, the investigation was conducted from 10th July 2021 to 15th September 2021. In detail, investigators of each city used the online questionnaire star platform (https://www.wjx.cn/) to distribute questionnaires one-on-one and face-to-face with people in their cities. Then, after the investigator entered the questionnaire number, respondents would complete the questionnaire by clicking on the link. If the respondents held the ability to think but were not able to act to answer the questionnaire, the investigator would help finish the questionnaire based on the offered answers by the participants.

### 2.2. Calculation of the minimum sample size

The minimum sample size was calculated using the following formula.


$$\begin{array}{l} n\;=\;\left[Z_{\;\alpha /2}^{2}\;p\;q\right]/\delta \; \end{array}$$

(25)

In the formula, n denotes the sample size, p denotes the estimated self-medication rate, q = 1-p, α = 0.05, Zα/2 = 1.96 ≈ 2, δ is the admissible error, δ = 0.1^*^p. According to the literature, the global population's self-medication rate ranges between 32.5 and 81.5%. Based on the formula, the minimum sample size is 831 if the smaller rate is used for sample size calculation. Given that 20% of the questionnaires may be invalid, the minimum number of questionnaires that should be distributed is 1,039 (26).

### 2.3. Inclusion criteria

This study first estimated the self-medication rate of Chinese residents. Then the researcher further analyzed the behavior of residents who had self-medication experience before, and the factors related to the consideration of medical staff's advice when self-medicating. The inclusion criteria for study's participants were as follows: (1) aged over 18 years old; (2) have purchased and used OTC drugs (that is, answer “yes” to the question of whether you have ever purchased and used OTC); (3) voluntarily participate in the research and fill in the informed consent form; (4) can complete the questionnaire independently or with the help of the investigator.

### 2.4. Exclusion criteria

**Figure 1:**
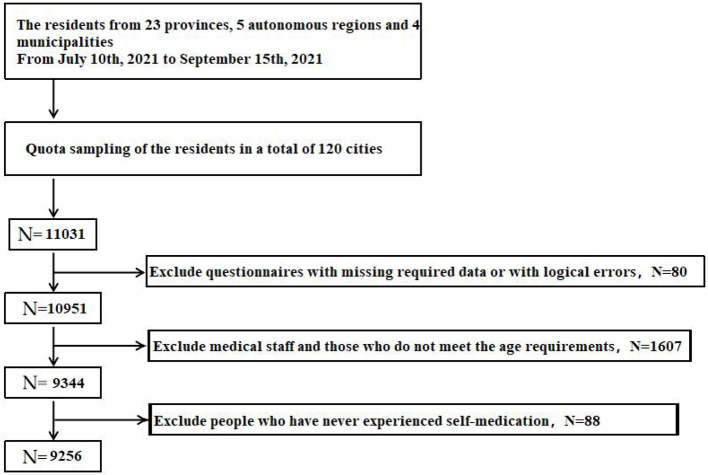
The flow-chart table.

The people with the following features were excluded from the study: (1) limited mobility, clouded in mind or have mental disorders; (2) participating in other similar research; (3) medical workers. Since the purpose of this study is to study the self-medication behavior of the public, medical workers were excluded from this study. Based on the above inclusion and exclusion criteria, a total of 9,256 subjects were finally included in this study, and the effective recovery rate of the questionnaire was 83.91% (9,256/11,031). As shown in Figure 1 below.

### 2.5. Research methods

#### 2.5.1. Research instruments

##### 2.5.1.1. General information questionnaire

A self-designed questionnaire was used for collecting respondents' general information which included gender, age, province, usual residence (urban/rural), education level, average income per family per month, marital status, the primary method of bearing current medical expenses, current occupational status (student/working/irregular/retired), and currently diagnosed chronic diseases.

##### 2.5.1.2. Partial questions from the questionnaire

Partial questions from the questionnaire whether the respondents have ever purchased and used OTC drugs on their own; and if so, the types of OTC drugs they choose and the factors to consider while purchasing.

This section included two entries (one single-choice and two multiple-choice). All items in this section were designed based on the current marketing and sales status of OTC drugs in China, relevant literature, and personal practice experience (27–37). Before the finalization of these questions, 2-round of expert consultation were conducted with two expert groups consisting of a panel of eight pharmacists with bachelor's degrees or above, working in secondary or tertiary hospitals.

The single question was set on “have you ever purchased and used OTC drugs on your own,” and those who answered “no” were excluded from this study. Following are two self-designed multiple-choice questions: First, the question “what types of OTC drugs have you ever purchased and used on your own,” include 10 following options: ① Antipyretic and analgesic drugs (e.g., paracetamol tablets); ② Digestive system drugs (e.g., ranitidine hydrochloride capsules); ③ Respiratory system drugs (e.g., aminogalactam tablets); ④ Vitamins and minerals (e.g., vitamin C tablets); ⑤ Antibacterial drugs (e.g., metronidazole cheek tablets) ⑥ External Drug for Skin (e.g., beclomethasone camphor cream); ⑦ Proprietary Chinese drugs (e.g., Xiao Chai Hu granules, Xiao Jian Zhong granules, Si Jun Zi Wan); ⑧ Gynecological drugs (e.g., miconazole nitrate suppositories) (only women had the option of “gynecological drugs” in the questionnaire); ⑨ Anti-allergy drugs (e.g., loratadine capsules); ⑩ and Others. The respondents could make multiple choices based on their experience.

The second multiple choice was “which of the following factors do you think is important for you to consider when purchasing OTC drugs on your own,” including 16 following options: ① Drug price; ② Drug efficacy; ③ Drug safety; ④ Drug taste; ⑤ Convenience of drug use; ⑥ Beauty of drug packaging; ⑦ Drug dosage form; ⑧ Brand awareness; ⑨ Availability of drug reimbursement by medical insurance; ⑩ Advice from doctors; ① Advice from pharmacists (there are no nurses and other medical staff in the questionnaire design, because they are far less comprehensive and familiar with pharmacy knowledge than clinicians and pharmacists, and are usually not the main providers of medication information for patients) (17); ② Suggestions from relatives and friends; ④ Personal experience; ⑤ Advertising; ⑥ After-sales service; ⑦ Corporate reputation. The number of available options for respondents ranged from 1 to 16. For each respondent, the option orders for both multiple-choice questions were randomized to reduce potential measurement bias.

##### 2.5.1.3. Short-form health literacy scale

In 2019, Duong et al. simplified the 47-item European Health Literacy Questionnaire (HLS-EU-Q47) to develop a short version of the health literacy scale (HLS-SF12), The scale was used to measure the health literacy (HL) of the respondents (38). In 2019, Duong et al. validated the reliability of the scale and investigated the health literacy of rural people in Vietnam (39), In 2021, Cheong et al. used the scale to study the health literacy and related factors of Filipino migrant workers (40), In 2022, Zhang et al. applied the scale to explore residents' attitudes toward vaccination (41), HLS-SF12 by Duong et al. had good reliability in Taiwan, China according to Duong TV's study (38). The total Cronbach's alpha for this scale was 0.85, The Cronbach's alphas for each dimension were 0.61 (health care), 0.67 (disease prevention), and 0.72 (health promotion), Item-scale convergent validity were 0.66 (health care), 0.69(disease prevention), 0.73 (health promotion), The validation factor analysis was used to test the structural validity of the scale for use in Taiwan, China, and the model fitness indicators were as follows RMSEA = 0.06, GFI = 0.97, AGFI = 0.95, CFI = 0.95, IFI = 0.95, NFI = 0.95, *X*^2^/df = 5.72 (38), All the above indicators of reliability and validity are qualified and meet the relevant criteria, which indicates that the HLS-SF12 has good reliability and validity in Taiwan, China and can be used to measure the health literacy of Chinese residents. The scale included three dimensions including health care, disease prevention, and health promotion, with 12 items, each rated on a 4-point scale (1 = very difficult, 2 = difficult, 3 = easy, 4 = very easy), using a formula to calculate a standardized HL index with a range of 0–50, with higher indices representing higher levels of health literacy. The calculation formula was, index = (mean−1) × (50/3), where the mean was the average of all items involved in each individual. In this scale, one was the minimum possible value of the mean (at this time the minimum value of the index is 0), three was the range of the mean, and 50 was the maximum value of the index. The higher the index indicated, the higher the level of health literacy of the survey respondents. In this study, the Cronbach's alpha of the short version of the health literacy scale was 0.940, and the Cronbach's alphas of the three subscales of health care, disease prevention, and health promotion were, respectively, 0.856, 0.860, and 0.868, all with good reliability. With reference to relevant literature, this study divided the respondents into two groups based on their health literacy: high group (above 33 points) and low group (33 points and below) (40, 42, 43).

##### 2.5.1.4. Ten-item Big Five inventory

The 10-item Big Five Inventory (BFI-10) was developed by Rammstedt and John. The scale is based on The Big Five personality model (44, 45), BFI-10 was used to measure the personality traits of the respondents. In 2021, Nikčević et al. applied the scale to explore the relationship between anxiety and depression of residents during the epidemic of COVID-19 (46), In 2021, Airaksinen et al. applied the scale to explore the influence of personality characteristics on the prevention behavior of COVID-19 (47), Carciofo et al. measured the Cronbach's alpha for each of the five dimensions of the Chinese version of the BFI-10. The Cronbach's alphas for each dimension were as follows: 0.752 (extraversion), 0.466 (agreeableness), 0.462 (consciousness), 0.628 (neuroticism), 0.525 (openness). The Cronbach's alphas for all five dimensions of the scale were >0.45, which is adequate for a two-item scale. The retest reliability for the five dimensions was 0.515–0.839 (45). All the above indicators of reliability are qualified and meet the relevant criteria, which indicates that the BFI-10 has favorable reliability. The scale consisted of 10 items in five dimensions: openness, agreeableness, neuroticism, consciousness, and extraversion, with each dimension containing two items. All questions on the scale were scored on a five-point likert scale, and each subscale was scored out of 10, with higher scores indicating a more pronounced personality trait. With reference to relevant literature, this study divided the respondents into two groups based on their personality traits: high groups (7–10 points) and low groups (6 points and below) (45, 48–50).

##### 2.5.1.5. Health effectiveness value visual analog scale

The most common tool for measuring health-related quality of life (HRQoL) is the European Five-Dimensional Health Scale (EQ-5D-5L), which was used to assess the health status of both patients and healthy individuals (51). The Health Effectiveness Value Visual Analog Scale (EQ-5D-VAS) was used to reflect people's health status by rating how good or bad their health was. In 2015, Hong et al. used the EQ-5D-VAS to examine factors associated with quality of life in Korean COPD patients (52). In 2019, Cha et al. applied the EQ-5D-VAS to compare differences in Americans' assessments of their own health in 2002 and 2017 (53), EQ-5D-VAS showed good metrological performance. EQ-5D-VAS was a component of the EQ-5D-5L that used the numbers between 0 and 100 to represent survey participants' self-rated health conditions, with 100 representing the best possible health condition and 0 the worst possible health condition (21). With reference to relevant literature, this study divided the respondents into two groups based on their EQ-5D-VAS scores: high groups (81–100 points) and low groups (80 points and below) (54, 55).

##### 2.5.1.6. New general self-efficacy scale

Self-efficacy of people was assessed using the New General Self-Efficacy Scale (NGSES), which Chen et al. modified from the General Self-Efficacy Scale (GSSE) (56). In 2012, Xiao et al. translated the scale into Chinese and validated its reliability and validity (57). In 2020, Tinaz et al. applied the scale to explore goal-directed behavior in Parkinson's patients (58). Xiao et al. measured the scale's Cronbach's alpha of 0.872, The split-half reliability coefficient of 0.811; The structural validity of the scale was tested by validation factor analysis, and the model fitness indicators were as follows: *X*^2^ = 0.028, *X*^2^/df = 1.683, PNFI = 0.659, GFI = 0.960, NFI = 0.941, CFI = 0.975, TLI = 0.965, RMSEA = 0.057 (57). All the above indicators of reliability and validity are qualified and meet the relevant criteria, which indicates that the Chinese version of the NGSES has good reliability and validity. The NGSES scale's Cronbach's alpha in this study was 0.935, demonstrating the scale's high level of internal consistency reliability. Eight numbers are on the scale (56, 59). The eight items were all scored using Likert 5-level scoring method. All items were scored positively, ranging from strongly disagree (1 point) to strongly agree (5 points); then scores of each item were accumulated to get the standard score, the higher the standard score, the better the self-efficacy. In this study, the following scores were used to grade the scale: a total scale score between 0 and 30 was considered low self-efficacy; a total score between 31 and 40 was considered high self-efficacy (60–62).

#### 2.5.2. Quality control

Before the start of the survey, the current study conducted two rounds of expert consultation and pre-investigation. The questionnaire was revised and finalized based on the suggestions of the pre-investigation and expert consultation. Then the selected investigators were trained on how to distribute questionnaires to respondents and registered their codes one-on-one and face-to-face. Every Sunday evening, the members of the research group communicated with the investigators to summarize, evaluate, and give feedback on the questionnaires they collected. After the questionnaires were collected, two people conducted back-to-back logic checks and data screening. If singular values are found during data analysis, the original questionnaire would be found and checked with the investigator before proceeding to the next step of the analysis.

#### 2.5.3. Statistical analysis

Statistical analyses were performed using SPSS 25.0 for Windows (SPSS, Inc., Chicago, IL, USA). The number and percentage of categorical variables were calculated. For normally distributed data, the mean and standard deviation were used for statistical description, and for non-normally distributed data, the median, and interquartile range were used for statistical description. All scale scores were transformed into dichotomous variables with reference to relevant literature, and categorical variables were expressed by frequency (composition ratio). Uni-variate binary logistic regression was used to identify the predictors of various pattern of purchased medication where the independent variable was the demographic and sociological characteristics of the respondents and the dependent variable was the type of various drugs; a uni-variate binary logistic regression was used to determine whether the respondents considered the advice of medical personnel as an important consideration when purchasing OTC drugs uni-variate analysis (63). Multivariate binary stepwise logistic regression (inclusion and exclusion criteria for variables were *P* = 0.05 and *P* = 0.10, respectively), was used to conduct a multi-factor analysis of whether the respondents considered the advice of medical personnel as an important consideration when purchasing OTC drugs, with a test level of α = 0.05. The independent variables for this part of the analysis included the demographic and sociological characteristics of the respondents and the health literacy scale, the dependent variable was whether the respondents considered the advice of medical personnel as an important consideration when purchasing OTC drugs on their own. The results of the variable analysis were subgroup analyzed and subgroups were classified according to gender (male, female), age (19–35, 35 years, or older), and usual residence (urban, rural).

#### 2.5.4. Ethical review

The current study has been approved by the Medical Ethics Committee of Jinan University (approval number: JNUKY-2021-018), and all participants were fully aware of the study and signed informed consent voluntarily.

## 3. Results

### 3.1. Common method variance test

Harman's single-factor test has suggested that 5 factors had eigenvalues >1, and the variance contribution of the first principal factor was 34.98% (not exceeding 40%). This indicated that there was no significant common method variance (64).

### 3.2. Basic information of respondents

This research included a total of 9,256 respondents, much larger than the calculated sample size of 1,039. Of the respondents, 46.34% were male (4,289), and 53.66% were female (4,967). 45.87% respondents were aged 19–35 (4,246), and 42.51% were aged 36–59 (3,935), with the rest 11.6% aged 60 and over (1,075). 60.2% of the respondents had received higher education (5,571), whilst 39.8% had not (3,685). The remaining demographic characteristics are shown in Table 1.

**Table 1:** Personal characteristics of the survey.

**Factors**		**Numbers**	**Percentage %**
**Gender**			
	Male	4,289	46.34
	Female	4,967	53.66
**Age (y)**			
	19–35	4,246	45.87
	36–59	3,935	42.51
	≥60	1,075	11.61
**Education**			
	Under college degree	3,685	39.8
	College degree or higher	5,571	60.2
**Monthly income per capita (CNY/USD)**			
	≤ ¥4,500 ( ≤ $631.3)	4,735	51.2
	≥¥4,501 (≥$631.4)	4,521	48.8
**Location**			
	Eastern China	4,722	51.0
	Midwest China	4,534	49.0
**Residential area**			
	Rural	2,582	27.9
	Urban	6,674	72.1
**Diagnosis of chronic disease**			
	No	7,358	79.5
	Yes	1,898	20.5

### 3.3. Self-purchase and use of OTC drugs

In the light of this investigation, 9,256 out of 9,344 people answered “yes” to the question—“have you ever bought and used OTC drugs on your own.” This represents that the self-medication rate of Chinese adults is about 99.06%. Survey respondents with self-medicated behaviors were chosen for further investigation in this research. Table 2 illustrates the number and proportion of these survey respondents in terms of each type of OCT drug purchased and used. Antipyretic analgesics and vitamins/minerals were the top two OTC drugs that had been purchased and utilized. Of the respondents, 58.57% had purchased and used antipyretics (5,421). The number for vitamins/minerals was 4,851, comprising 52.41%. Except for the “other” option, the number of respondents who had bought and used anti-allergy and gynecological medicines was relatively small. Only 1,322 respondents used anti-allergy medication without a prescription. Eight hundred ninety-eight respondents had an experience buying and using gynecological medicines, reaching 22.00% of all women respondents. Information on variable assignment for univariate and multivariate analysis is provided in Supplementary Table 1.

**Table 2:** Types and proportion of OTC drugs purchased and used by respondents.

**Types**	**Numbers**	**Percentage %**
NSAIDs	5,421	58.57
Vitamins and minerals	4,851	52.41
External drug for skin	3,480	37.60
Digestive system medication	3,289	35.53
Antibacterials	3,219	34.78
Chinese patent medicine	2,435	26.31
Respiratory system medication	1,649	17.82
Antiallergic medication	1,322	14.28
Gynecological medication^*^	898	22.0 (in female respondents)
Other	167	1.80

^*^The option “gynecological medication” is only shown on questionnaires for female respondents.

### 3.4. Correlation between the sociodemographic characteristics of survey respondents and the types of self-purchased and used OTC drugs

Adopting binary logistic regression, a univariate analysis of variance was conducted to examine the relationship between the sociodemographic characteristics of the survey respondents and the types of OTC drugs purchased and used without prescription. Table 3 demonstrates the results of the univariate logistic regression, adopting “whether or not the respondents had self-purchased antipyretic analgesics and antibacterial drug ever” as the dependent variable and “the sociodemographic characteristics of the respondents” as the independent variable. The results of the univariate logistic regression on the relationship between other types of drugs and the sociodemographic characteristics of the respondents are presented in Supplementary Tables 2–8.

**Table 3:** One-way binary logistic regression analysis results of NSAIDs, antibacterial drugs, and social demographic characteristics.

**Drug type**	**Variable**	β	***P***	**OR**	**95% CI (Lower)**	**95% CI (Upper)**
**NSAIDs**	**Gender (The control group is male)**					
	Female	−0.174	**< 0.001**	0.840	0.773	0.913
	Location within 3 months (The control group is eastern China)					
	Midwest China	−0.117	**0.006**	0.890	0.819	0.967
	Chronic disease or not (The control group is No)					
	Yes	0.161	**0.002**	1.175	1.059	1.303
	Residential area (The control group is rural)					
	Urban	−0.113	**0.005**	0.875	0.798	0.960
	Self-assessment of health (The control group is low score group)					
	High score group	−0.136	**0.002**	0.873	0.802	0.950
	Monthly income per capita (The control group is ≤ ¥4,500)					
	≥¥4,501	0.040	0.349	1.040	0.958	1.130
	Age range (The control group is 19–35 years old)					
	36–59	0.065	**0.146**	1.067	0.978	1.165
	≥60	0.405	**< 0.001**	1.449	1.303	1.725
	Education (The control group is under college degree)					
	College degree or higher	−0.190	**< 0.001**	0.827	0.760	0.900
Antibacterial	Gender (The control group is male)					
	Female	0.068	0.119	1.071	0.983	1.167
	Location within 3 months (The control group is eastern China)					
	Midwest China	−0.034	0.437	0.967	0.887	1.053
	Chronic disease or not (The control group is No)					
	Yes	0.333	**< 0.001**	1.395	1.258	1.548
	Residential area (The control group is rural)					
	Urban	0.134	**0.006**	1.143	1.038	1.259
	Self-assessment of health (The control group is low score group)					
	High score group	−0.006	0.889	0.994	0.912	1.085
	Monthly income per capita (The control group is ≤ ¥4,500)					
	≥¥4,501	0.009	0.837	1.009	0.926	1.099
	Age range (The control group is 19–35 years old)					
	36–59	0.254	**< 0.001**	1.289	1.177	1.412
	≥60	0.153	**0.033**	1.165	1.012	1.341
	Education (The control group is under college degree)					
	College degree or higher	0.025	0.576	1.025	0.939	1.119

The bold values indicate that the value is statistically significant.

### 3.5. Descriptive statistics on driving factors for survey respondents in purchasing OTC drugs

The percentage of survey respondents who considered a physician advice a decisive factor in purchasing OTC drugs was 73.60% (6,512). Five thousand two hundred eleven respondents took the pharmacist's advice into account when buying OTC drugs, reaching a proportion of 56.30%. The present research refers to the methods used in other studies (1, 65), whereby the professional advice from physicians and that from pharmacists were merged into the medical staff's advice. Therefore, the percentage of survey respondents who considered medical staff's advice (physician's or pharmacist's advice) an important factor was 86.20% (7,979). A detailed description can be found in Table 4.

**Table 4:** Descriptive statistics of important considerations for OTC drug purchase.

**Option**	**Numbers**	**Percentage %**
Suggestions from medical personnel	7,979	86.20
Doctor's suggestions	6,812	73.60
Pharmacist's suggestions	5,211	56.30
Drug safety	5,901	63.69
Drug efficacy	5,492	59.28
Suggestions from relatives and friends	4,816	52.00
Personal experience	4,457	48.11
Drug price	3,473	37.49
Reimbursement with medical insurance	2,641	28.51
Brand awareness	2,332	25.17
Enterprise reputation	1,865	20.13
Dosage form	1,719	18.55
Convenience of taking medicine	1,688	18.22
After-sale service	1,062	11.46
Advertising	878	9.48
Drug taste	871	9.40
Drug packaging	445	4.80

### 3.6. The scores from health literacy scale-the short version, Big Five personality scale, EQ-5D-VAS, and self-efficacy scale

The scores of the short version of the health literacy scale, the Big Five personality scale, EQ-5D-VAS, and the self-efficacy scale were shown in Table 5. Since the scores of each scale did not meet the standard of the normal distribution, the median, upper quartiles, and lower quartiles were used to describe the central tendency and dispersion of the scores of each scale. In general, 5,942 people (64.20%) had high health literacy (i.e., the score of the short version of the health literacy scale was higher than 33 points); people of high extroversion, high agreeableness, high consciousness, high neuroticism, and high openness were 3,349 (36.18%), 5,182 (55.99%), 4,757 (51.39%), 2,257 (24.38%), and 3,565 (38.52%), respectively; it should be clarified that the people who got more than 6 in Big Five Personality Scale were considered to be typical in the five personality traits. Also, there were 5,460 people (58.99%) with high EQ-5D-VAS scores and 4,057 people (43.83%) with high self-efficacy scores.

**Table 5:** Scores of HLS-SF12, BFI-10, EQ-5D-VAS, and NGSES of respondents.

	**Number of items**	**Score range**	**K–S test**	**K–S test significance**	**Median**	**Lower quartile–Upper quartile**	**Number and proportion of high score group**	**Number and proportion of low score group**
HLS-SF12	12	0–50	0.208	**< 0.001**	33.33	30.56–37.50	5,942 (64.20%)	3,314 (35.80%)
**BFI-10**								
Extraversion	2	2–10	0.203	**< 0.001**	6	5–7	3,349 (36.18%)	5,907 (63.82%)
Agreeableness	2	2–10	0.187	**< 0.001**	7	6–8	5,182 (55.99%)	4,074 (44.01%)
Conscientiousness	2	2–10	0.200	**< 0.001**	7	6–8	4,757 (51.39%)	4,499 (48.61%)
Neuroticism	2	2–10	0.217	**< 0.001**	6	5–6	2,257 (24.38%)	6,999 (75.62%)
Openness	2	2–10	0.221	**< 0.001**	6	6–7	3,565 (38.52%)	5,691 (61.48%)
EQ-5D-VAS	1	0–10	0.148	**< 0.001**	84	73–96	5,460 (58.99%)	3,796 (41.01%)
NGSES	8	8–40	0.135	**< 0.001**	29	24–32	4,057 (43.83%)	5,199 (56.17%)

The bold values indicate that the value is statistically significant.

### 3.7. The result of univariate binary logistic regression analysis

A univariate analysis was conducted using binary logistic regression to examine whether “medical staff” was an important consideration when purchasing OCT drugs. The results showed statistically significant differences (*p* < 0.05) in the respondents' probability of considering medical staff's advice as a decisive factor when purchasing OTC drugs concerning gender, Location within 3 months, age, diagnosis with chronic disease or not, extroversion rating, agreeableness rating, rigor rating, openness rating, health literacy rating, EQ-5D-VAS score rating, and self-efficacy rating. Detailed information can be seen in Table 6.

**Table 6:** Single factor analysis for whether survey respondents consider medical staff's advice as an important factor when purchasing OTC drugs.

**Variable**	**β**	***P***	**OR**	**95% CI (Lower)**	**95% CI (Upper)**
**Gender (The control group is male)**					
Female	0.183	**0.002**	1.201	1.067	1.352
**Location within 3 months (The control group is eastern China)**							
Midwest China	0.137	**0.024**	1.146	1.018	1.291
**Residential area (The control group is rural)**					
Urban	0.106	0.110	1.111	0.976	1.265
**Monthly income per capita (The control group is** **≤**¥**4,500)**							
≥¥4,501	−0.045	0.460	0.956	0.850	1.076
**Age range (The control group is 19–35 years old)**							
36–59	0.132	**0.039**	1.142	1.007	1.294
≥60	0.165	0.103	1.180	0.967	1.438
**Education (The control group is under college degree)**					
College degree or higher	−0.032	0.605	0.969	0.858	1.093
**Chronic disease or not (The control group is No)**					
Yes	−0.173	**0.026**	0.841	0.723	0.980
**Extraversion (The control group is low score group)**					
High score group	0.335	**< 0.001**	1.398	1.230	1.589
**Agreeableness (The control group is low score group)**					
High score group	0.708	**< 0.001**	2.031	1.801	2.290
**Conscientiousness (The control group is low score group)**							
High score group	0.571	**< 0.001**	1.771	1.569	1.998
**Neuroticism (The control group is low score group)**							
High score group	0.133	0.064	0.143	0.992	1.316
**Openness (The control group is low score group)**							
High score group	0.403	**< 0.001**	1.497	1.318	1.700
**Health literacy (The control group is low in health literacy)**							
High	0.348	**< 0.001**	1.417	1.257	1.597
**EQ-5D-VAS score (The control group is low score group)**					
High score group	0.293	**< 0.001**	1.341	1.191	1.510
**NGSES score (The control group is low score group)**					
High score group	0.528	**< 0.001**	1.695	1.497	1.920

The bold values indicate that the value is statistically significant.

### 3.8. Multi-factor binary stepwise logistic regression analysis of whether medical professionals' advice is an important consideration when purchasing OTC drugs

A multivariate binary stepwise logistic regression analysis was conducted, taking whether respondents took the advice of medical personnel as an important consideration when purchasing OTC drugs as the dependent variable, and the demographic and sociological characteristics of respondents and the score grading of each scale (all screened by univariate analysis, and related to the dependent variable) as the independent variable. The established model Ominbus test *P* < 0.001, −2 log likelihood value of 7,174.086, Hosmer-Lemeshaw test *P* = 0.514 > 0.05, indicating the model quality was good.

According to multifactorial binary stepwise logistic regression, gender, place of residence, the presence of chronic diseases, the Big Five personality ratings of agreeableness, rigor, neuroticism, and openness, health literacy score rating, EQ-5D-VAS scale score rating, and self-efficacy score rating were linked to whether survey respondents considered medical professionals' advice as an important consideration when purchasing OTC drugs. Women were more likely to consider medical advice as an important factor than men (OR = 1.160, 95% CI 1.028–1.309, *P* < 0.05); respondents in central and western China were more likely to consider medical advice as an important factor than those living in eastern China (OR = 1.153, 95% CI 1.022–1.300, *P* < 0.05); respondents with chronic diseases were less likely to consider medical advice as an important factor than those without chronic diseases (OR = 0.767, 95% CI 1.0.653–0.900, *P* < 0.05); and respondents with high habitability were more likely to consider medical advice as an important factor than those with low habitability (OR = 1.668, 95% CI 1.464–1.900, *P* < 0.001); compared to those with low rigor, respondents with high rigor were more likely to consider medical staff advice as an important factor (OR = 1.336, 95% CI 1.172–1.523, *P* < 0.001); compared to those with low neuroticism, respondents with high neuroticism were more likely to consider medical staff advice as an important consideration (OR = 1.163, 95% CI 1.005–1.346, *P* < 0.05); and respondents with high openness were more likely to have medical staff advice as an important consideration compared to those with low openness (OR = 1.145, 95% CI 1.000–1.310, *P* < 0.05). Compared to those with low health literacy, respondents with high health literacy were more likely to consider medical staff advice as an important consideration (OR = 1.312, 95% CI 1.151–1.495, *P* < 0.001); compared to those with low EQ-5D-VAS subgroup, respondents with high EQ-5D-VAS subgroup were less likely to consider medical staff advice as an important consideration (OR = 1.152, 95% CI 1.015–1.308, *P* < 0.05), and respondents in the high self-efficacy group were more likely to consider medical staff advice as an important consideration compared to the low self-efficacy group (OR = 1.330, 95% CI 1.160–1.524, *P* < 0.001), (see Table 7) for details.

**Table 7:** Stepwise multivariate binary logistic regression results of whether the respondents take the suggestions of medical personnel as an important consideration when purchasing OTC drugs.

**Variable**	**β**	**SE**	**Wald *χ^2^***	***P***	**OR**	**95% CI (Lower)**	**95% CI (Upper)**
**Gender (The control group is male)**							
Female	0.148	0.062	5.763	**0.016**	1.160	1.028	1.309
**Location within 3 months (The control group is eastern China)**							
Midwest China	0.142	0.062	5.330	**0.021**	1.153	1.022	1.300
**Chronic disease or not (The control group is No)**							
Yes	−0.265	0.082	10.537	**0.001**	0.767	0.653	0.900
**Agreeableness (The control group is low score group)**							
High score group	0.511	0.066	59.138	**< 0.001**	1.668	1.464	1.900
**Conscientiousness (The control group is low score group)**							
High score group	0.290	0.067	18.774	**< 0.001**	1.336	1.172	1.523
**Neuroticism (The control group is low score group)**							
High score group	0.151	0.075	4.108	**0.043**	1.163	1.005	1.346
**Openness (The control group is low score group)**							
High score group	0.135	0.069	3.863	**0.049**	1.145	1.000^*^	1.310
**Health literacy (The control group is low in health literacy)**							
High	0.271	0.067	16.474	**< 0.001**	1.312	1.151	1.495
**EQ-5D-VAS score (The control group is low score group)**							
High score group	0.141	0.065	4.777	**0.029**	1.152	1.015	1.308
**NGSES score (The control group is low score group)**							
High score group	0.285	0.070	16.770	**< 0.001**	1.330	1.160	1.524

^*^This value retains three decimal places as other values. The bold values indicate that the value is statistically significant.

## 4. Discussion

### 4.1. Self-medication rate of Chinese residents

Pharmacies and community health centers have been facilitating residents' access to OTC drugs. The results of this study indicated that the self-medication rate among Chinese adults was as high as 99.06% (66). Previous research has found that the self-medication rate for US residents was ~77% in 2012. In 2016, the self-medication rate for Iran residents was about 68% (67), and that for Indian residents was around 31% (68). In 2021, a self-medication rate of ~88.2% was found among Thailand residents (2, 69). In 2018, Lei et al. explored the self-medication practices and relevant factors among residents in Wuhan, China, and they found about 45.40% of the self-medication rate of Wuhan residents (70). The present study was conducted after the breakout of the COVID-19 pandemic, at a time when the pandemic in mainland China was in a sporadic outbreak phase. During this period, the epidemic was disseminated in mainland China including Beijing, Henan, and Jiangsu. There were many people self-quarantined, while several hospitals had introduced policies that patients should be required to provide a negative nucleic acid test result taken within 24 or 48 h to gain entry admission to hospitals (71). Thereby, access to hospitals became difficult, and residents were more likely to self-medicate by purchasing appropriate medicines online or offline when they feel sick. The COVID-19 pandemic could be a major reason for the increase in self-medication rates among Chinese residents.

### 4.2. Types and reasons of OTC drugs purchased by Chinese residents

The two most common types of OTC drugs purchased and used by survey respondents in this study were antipyretic analgesics (5,421 respondents, 58.57%) and vitamins/minerals (4,851 respondents, 52.41%). This result is consistent with the most common self-medications used by residents of Australia and Ethiopia in previous studies (72, 73). Many studies have found that antipyretic analgesics are currently the most commonly used self-medication drugs in several countries (74). Antipyretic analgesics can not only relieve the symptoms of COVID-19, but also relieve the headache and fever symptoms of common diseases such as cold, dysmenorrhea, gout, flu, etc., China Epidemic Prevention and Control Policy requires outbreak areas to restrict the purchase of antipyretic analgesics. The drugs are commonly used and residents will purchase reserves (75). This seems evident from the proportion of respondents who chose to purchase vitamins/minerals that the majority valued their own and their family's health and nutrition. A similar conclusion has been found in 2018, which suggested that more than half of residents in countries, such as Poland (70%) and Canada (57%), would purchase vitamins/minerals (76).

### 4.3. The relationship between sociodemographic characteristics and the likelihood of residents purchasing antipyretic analgesics

OTC antipyretic analgesics mainly included non-steroidal antipyretic and anti-inflammatory drugs, such as aspirin tablets and ibuprofen tablets. As suggested, males were more likely to self-purchase and use antipyretic analgesics, which is probably related to males' higher physical workload and less frequency of medical consultations and visits (77, 78). Residents in eastern China were more likely to have self-purchased such drugs. This tendency could be associated with the lower density of hospitals and pharmacies located in the central and western regions and a comparatively higher development level of primary health care resource allocation in the east than in the central and western China (79). Regarding chronic diseases, this present research has a similar finding to a study conducted in 2018, which found that patients suffering from chronic conditions, particularly chronic pain, used antipyretic analgesics twice as often as those without chronic painful conditions (80). Those with permanent residence in rural areas were more likely to self-purchase this type of OTC drugs. This was probably related to the fact that residents living in rural areas had less access to healthcare resources than in urban areas (80, 81). People aged 60 years and over were more likely to purchase OTC antipyretic analgesics, possibly due to the high prevalence of chronic pain in this age group (82, 83). People who had not been to universities degree were more likely to buy antipyretic analgesics, possibly because those with higher education were equipped with more medical-related knowledge and were, therefore, more cautious when deciding to purchase medication on their own (84). Furthermore, the use of antipyretic analgesics could be relevant to one's health condition, whereby people in poorer conditions are more prone to purchase this type of drug (84, 85).

### 4.4. The relationship between sociodemographic characteristics and the likelihood of residents purchasing antibiotics

It is pervasive today for pharmacies in China to sell prescription antimicrobials without a prescription, and how to reduce the abuse of antibiotics among the population is consistently an issue worthy of consideration (86). Most of the antimicrobials in OTC drugs were topical antimicrobials (e.g., erythromycin ointment), which can be used for skin infections (e.g., abscess sores) and diseases (e.g., periodontitis). People with chronic illnesses were more inclined to purchase this type of medicine, and the demand for OTC antimicrobials is higher in this group (87). People with permanent residence in towns and cities were more likely to buy antimicrobials, as this type of resident has more opportunities to buy (88), given a large number of widely distributed pharmacies in towns and cities (89). Meanwhile, people aged 36–59, 60, and over were more likely to purchase antimicrobials, which was associated with a gradual rise in the use of this type of drugs with aging (77, 82).

### 4.5. Current status of Chinese residents taking the advice of medical staff as an important consideration when purchasing OTC drugs

The study reveals that in China the fact that the number of people who followed doctors' advice was higher than that of pharmacists was related to the fact that there were more doctors than pharmacists and Chinese residents had higher trust in doctors than in pharmacists (90). From the above case, it could be seen that the traditional concept that doctors took charge of diagnosing patients' medical conditions while pharmacists only dispense drugs based on prescriptions is still widespread in China (91). Additionally, the study shows that in China, on the one hand, the inpatient rarely had a chance to communicate with pharmacists directly; on the other hand, there were no pharmacists in primary medical institutions (such as village clinics). In this study, 86.20% of the respondents who purchased OTC drugs took medical staff's advice as an important consideration, which indicates that advice from medical staff was one of the important considerations for respondents to purchase OTC medications. This finding is consistent with the results from a 2012 survey of residents of Tallinn, Estonia (92) and a 2012 survey in the Iraqi context (93). This result is positive because the self-administration of OTC medications without reliable medication advice from a healthcare provider is likely to increase the risk of adverse drug events.

### 4.6. Sociodemographic characteristics are related to the factors that Chinese residents' taking medical staff suggestions as important consideration when purchasing OTC drugs

It is demonstrated by the current research that women had a higher frequency of self-medicine than men and they were more likely to consider medical practitioner's advice as an important consideration when purchasing OTC drugs, which consistent with previous conclusions (94). Also, the allocation level of medical resources in central and western China was lower than that of eastern China, further leading to a lower number of medical personnel and medical levels in central and western China than in eastern China. At the same time, there was fewer hospitals and pharmacies in the central and western China (95). In such a case, when self-medicating, residents in central and western China were found to be less likely to consider medical staff's advice as an important consideration than residents of eastern China. At present, China had a large population of chronic diseases and a large demand for OTC drugs (96). Those chronic patients who were mostly the elderly normally required long-term medication and may experience unstable changes in their medical conditions. For them, they were more likely to take the medical staff's advice as an important consideration. A Korean study in 2013 investigated the factors that affect the expenditure of OTC drugs and the study showed that the elderly with chronic diseases were exposed to less drug-related knowledge, and thus during the process of taking medicines tended to follow advice of the medical staff (97).

### 4.7. Self-efficacy, health literacy, health status, and personality traits are related to the factors that Chinese residents' taking medical staff suggestions as important consideration when purchasing OTC drugs

Besides, personal traits were found to largely determine the healthy behavior of patients (98). In the field of health psychology, the Big Five personality theory was widely used in different studies. This study indicated that people with high agreeableness, high openness, high conscientiousness, high extraversion, and high neuroticism were more likely to take medical staff's advice as an important consideration (*P* < 0.05). The agreeableness stemmed from socially pleasant behavior, and individuals whose scores were high in this regard would be generous, trusting, and participative (99), and such groups may have higher levels of trust in medical staff; People with high openness have strong critical thinking skills, were good at analyzing problems and have a thirsty for knowledge (100); these groups of people were more likely to consider the suggestions of medical staff and make choices that suit their physical conditions best (101). Consciousness was related to a cautious or vigilant mind state. People with this personal trait usually desired to do one thing well. Therefore, they would make decisions after the comprehensive consideration of many aspects. Neuroticism was about the way individuals deal with negative emotions and behaviors in a complicated situation. People scoring high on this trait were more likely to be worried and would concern about the side effects of the OTC drugs adverse (100). Therefore, they were also the group of people who would regard medical practitioners' advice as an important consideration (99). In 2015, Kamran et al. investigated Iranian residents and demonstrated a correlation between the behavior of self-medication and health literacy and health conditions (102). Health literacy refers to the ability of individuals to obtain and understand general health information and services and based on what they know, to maintain and promote their health (103). People with high health literacy would identify their health problems in a general way and are more likely to consider the advice of medical staff when purchasing OTC medicines (104). Also, people with higher self-health scores paid more attention to their health issues and would more consideration the advice of medical staff when purchasing OTC medicines (105). In 2019, a study by Favel Mondesir showed that self-efficacy also played a positive role in residents' process of deciding and taking drugs (106). High self-efficacy can also promote residents to change their lifestyles based on their illness (107). There was also a high possibility for those people to follow the advice of the medical staff when buying OTC medicines.

### 4.8. Recommendations

A resident's reasonable self-medicating behavior is closely linked with advice from medical staff. However, collective actions from the government, drug manufacturers, and medical personnel are needed for regulating residents' self-medication. To ensure the legal sales of non-prescription drugs, authorities such as the State Drug Administration should further formulate and improve relevant laws and regulations to regulate offline and online drug sales, and guide consumers to self-medicate scientifically and rationally. The government in China should also bring further punishment for those who sell fake and inferior drugs, introduce more channels for residents to purchase OTC drugs, and encourage the construction of Internet hospitals and Internet pharmacies.

For drug manufacturers, since the drug instruction is a valuable tool to inform the public about drug-taking information, they can further clarify the way to take drugs and the possible side effects within the drug instructions and increase the readability of the instructions by adding pictures or explaining understandably (3). The medical personnel including clinicians, pharmacists, and so on, could enhance their professional knowledge and communication skills and put themselves in the shoes of residents who need to buy drugs for treatment. Those medical staff ought to provide scientific medical information and medical advice and help popularize drug-related knowledge to residents. They should also firmly reject malicious provisions and the promotion of false drug information. It is also suggested that the medical staff should take an active role in offering online (Purchase OTC drugs through the Internet) [Online hospitals, pharmacies, software, or websites (Meituan, Alipay, JingDong Online Pharmacy, Taobao Online Drugstore, etc.), and deliver them to your door by courier or delivery person] and offline (Residents go to the hospital, pharmacy or clinic to buy OTC drugs) drug consultation and provide guidance and assistance to drug users (108, 109). For those ordinary people who need medication information, they should gain the related information about OTC drugs through standardized channels. When listening to suggestions from different people, the residents should take the drugs according to their health conditions and corresponding symptoms. More attention from the residents should be paid to reading drug instructions carefully, especially in terms of the drugs' indications, side effects, and how to take drugs (110).

### 4.9. Research strengths and limitations

One strength of the current study is that the results of the research are based on the analysis from a nationwide cross-sectional survey in 2021, which means the findings are generally extensive and representative. Besides, this investigation analyzed the residents' non-prescription drug purchases from the aspects of personality, self-efficacy, health literacy, and health-related quality of life, which could further increase the application value of the health belief and self-efficacy theories. At the same time, the practical contributions are made by the targeted suggestions for different groups regarding how to conduct self-medicating supervision and how to better spread health-related knowledge to the public.

This study has some limitations. First, the data are solely collected from self-reported questionnaires, the result of which may not be totally reliable and could be affected by respondents' social expectations, self-report errors, and poor memory. Second, this study used a cross-sectional design that did not allow for causal inferences. Third, the mechanism of how self-assessed health status, health literacy, personality characteristics, and self-efficacy work together to affect respondents' consideration for purchasing OTC drugs needs further research. Fourth, the participants in this study are all in China, so the conclusion cannot be generalized to other countries. This limitation could be addressed in future studies by ensuring participants hold more diverse backgrounds. In addition, the behavioral characteristics of residents and the important factors considered in OTC drug purchases are likely to change in the next few years.

## 5. Conclusion

The vast majority of Chinese adults had the tendency to self-purchase and used OTC medicines. The two most commonly purchased OTC medicines were antipyretic analgesics and vitamins/minerals. The important considerations for residents when purchasing OTC medicines included medical staff's recommendations, drug safety, drug efficacy, and so on. In addition, whether China's residents would take medical staff's advice for consideration when buying OTC medicines was influenced by the residents' sociodemographic characteristics, health literacy, health self-assessment scores, self-efficacy, and personality. The suggestion was when taking OTC medicines, the residents should carefully listen to the advice of professional medical staff, and self-medicated based on their own medical and physical conditions.

## Data availability statement

The raw data supporting the conclusions of this article will be made available by the authors, without undue reservation.

## Ethics statement

The studies involving human participants were reviewed and approved by Institutional Review Committee of Ji'nan University, Guangzhou, China (JNUKY-2021-018). The patients/participants provided their written informed consent to participate in this study.

## Author contributions

PG, XS, and YW: conception and design. WY: acquisition of data. PG, QL, MD, and YN: analysis and interpretaion of data. PG, QL, and XH: drafting of the manuscript. PX, YB, HM, DL, ZZ, XS, JZ, and YW: critical revision of the manuscript for important intellectual content. PG and QL: statistical analysis. LY and YW: obtaining funding and supervision. YW: administrative, technical, or material support. YN: others: language polish. All authors contributed to the article and approved the submitted version.

## References

[1] Marquez GE Torres VE Sanchez VM Gramajo AL Zelaya N Peña FY. Self-medication in ophthalmology: a questionnaire-based study in an Argentinean population. Ophthalmic Epidemiol. (2012) 19:236–41. 10.3109/09286586.2012.689076 22775280

[2] Chautrakarn S Khumros W Phutrakool P. Self-Medication with OTC medicines among the working age population in metropolitan areas of Thailand. Front Pharmacol. (2021) 12:726643. 10.3389/fphar.2021.726643 34456738 PMC8385363

[3] Cho JH Lee TJ. The factors contributing to expenditures on OTC drugs in South Korea. Value Health Reg Issues. (2013) 2:147–51. 10.1016/j.vhri.2013.01.010 29702844

[4] Wang H Yao F Wang H Wang C Guo Z. Medication adherence and influencing factors among patients with severe mental disorders in low-income families during COVID-19 outbreak. Front Psychiatry. (2022) 12:799270. 10.3389/fpsyt.2021.799270 35115971 PMC8803649

[5] Takata M Wada Y Iwasawa Y Kumazawa M Shimokawa KI Ishii F. Criteria for the selection of switch OTC drugs based on patient benefits, efficacy, and safety [II]: comparing the physicochemical and pharmaceutical properties of brand-name and switch OTC terbinafine hydrochloride cream. Drug Discov Ther. (2018) 12:248–53. 10.5582/ddt.2018.01043 30224597

[6] Gilson AM Stone JA Reddy A Chui MA. Exploring how pharmacists engage with patients about OTC medications. J Am Pharm Assoc. (2019) 59:852–6. 10.1016/j.japh.2019.08.001 31501006 PMC6861682

[7] Bo S Chen G Ning L. The reform and enlightenment of FDA's OTC monograph system. Chin J New Drugs. (2022) 31:1157–62.

[8] Hui Z. The OTC Drug Catalogue Will be Dynamically Managed. Xi'an Daily (2004).

[9] Suggestions on the “Fourteenth Five Year” Development Plan of China's OTC Drug Industry. (2021). Available online at: https://www.cnma.org.cn/h-nd-541.html (accessed December 2, 2021).

[10] Badiger S Kundapur R Jain A Kumar A Pattanshetty S Thakolkaran N. Self-medication patterns among medical students in South India. Australas Med J. (2012) 5:217–20. 10.4066/AMJ.2012.1007 22848313 PMC3395275

[11] Chan V Tran H. Purchasing OTC medicines from Australian pharmacy: what do the pharmacy customers value and expect? Pharm Pract. (2016) 14:782. 10.18549/PharmPract.2016.03.782 27785164 PMC5061520

[12] Coombes H Cooper RJ. Staff perceptions of prescription and OTC drug dependence services in England: a qualitative study. Addict Sci Clin Pract. (2019) 14:41. 10.1186/s13722-019-0170-4 31718716 PMC6852756

[13] Amaha MH Alemu BM Atomsa GE. Self-medication practice and associated factors among adult community members of Jigjiga town, Eastern Ethiopia. PLoS ONE. (2019) 14:e0218772. 10.1371/journal.pone.0218772 31251745 PMC6599130

[14] National Regulation Database. Medical Law. National Regulation Database. Available online at: https://web.archive.org/web/20210117200211/https://law.moj.gov.tw/LawClass/LawAll.aspx?PCode=L0020021 (accessed June 24, 2019).

[15] National Regulation Database. Personnel Regulations for Medical Personnel. National Regulation Database. Available online at: https://web.archive.org/web/20200807011116/https://law.moj.gov.tw/LawClass/LawAll.aspx?pcode=L0020009 (accessed June 24, 2019).

[16] National Regulation Database. Measures for Practice Registration and Continuing Education of Medical Personnel. National Regulation Database. Available online at: https://web.archive.org/web/20201129202451/https://law.moj.gov.tw/LawClass/LawAll.aspx?PCODE=L0020181 (accessed June 24, 2019).

[17] Qianghong P Zhu C Jiang X Zhang F. Investigation on the cognition of medical staff to the work of clinical pharmacists. Prescript Drugs China. (2021) 19:42–43.

[18] Grebenar D Nham E Likic R. Factors influencing pharmacists' OTC drug recommendations. Postgrad Med J. (2020) 96:144–8. 10.1136/postgradmedj-2019-136969 31562196

[19] Bandura A. Self-efficacy: The Exercise of Control. New York, NY: Freeman (1997).

[20] Roundtable Roundtable on Health Literacy Board Board on Population Health and Public Health Practice Institute Institute of the Medicine. Facilitating State Health Exchange Communication Through the Use of Health Literate Practices: Workshop Summary. National Academies Press (2012).

[21] Ping W Zheng J Niu X Guo C Zhang J Yang H. Evaluation of health-related quality of life using EQ-5D in China during the COVID-19 pandemic. PLoS ONE. (2020) 15:e0234850. 10.1371/journal.pone.0234850 32555642 PMC7302485

[22] Rothmann S Coetzer EP. The big five personality dimensions and job performance. SA J Indust Psychol. (2003) 29:a88. 10.4102/sajip.v29i1.88

[23] *2021 China Family Health Index General Report*. Available online at: https://www.cfnews.org.cn/newsinfo/2685237.html (accessed April 19, 2022).

[24] National Bureau of Statistics. Bulletin of the Seventh National Population Census. National Bureau of Statistics (2022). Available online at: http://www.stats.gov.cn/search/s?qt=%E7%AC%AC%E4%B8%83%E6%AC%A1%E4%BA%BA%E5%8F%A3%E6%99%AE%E6%9F%A5 (accessed November 5, 2021).

[25] Samo AA Sayed RB Valecha J Baig NM Laghari ZA. Demographic factors associated with acceptance, hesitancy, and refusal of COVID-19 vaccine among residents of Sukkur during lockdown: a cross sectional study from Pakistan. Hum Vaccin Immunother. (2022) 18:2026137. 10.1080/21645515.2022.2026137 35103572 PMC8993050

[26] Kassie AD Bifftu BB Mekonnen HS. Self-medication practice and associated factors among adult household members in Meket district, Northeast Ethiopia, 2017. BMC Pharmacol Toxicol. (2018) 19:15. 10.1186/s40360-018-0205-6 29636092 PMC5894137

[27] Brabers AE Van Dijk L Bouvy ML De Jong JD. Where to buy OTC medications? A cross-sectional survey investigating consumers' confidence in OTC (OTC) skills and their attitudes towards the availability of OTC painkillers. BMJ Open. (2013) 3:e003455. 10.1136/bmjopen-2013-003455 24071460 PMC3787475

[28] Barrenberg E Garbe E. Use of OTC (OTC) drugs and perceptions of OTC drug safety among German adults. Euro J Clin Pharmacol. (2015) 71:1389–96. 10.1007/s00228-015-1929-5 26300207

[29] Hedenrud T Andersson Sundell K Martinsson J Håkonsen H. Attitudes towards sales and use of OTC drugs in Sweden in a reregulated pharmacy market: a population-based study. Int J Pharm Pract. (2019) 27:17–24. 10.1111/ijpp.12457 29687513

[30] Sánchez-Sánchez E Fernández-Cerezo FL Díaz-Jimenez J Rosety-Rodriguez M Díaz AJ Ordonez FJ. Consumption of OTC drugs: prevalence and type of drugs. Int J Environ Res Public Health. (2021) 18:5530. 10.3390/ijerph18115530 34064096 PMC8196755

[31] El-Gamal F Alsahafi BA Alsammak I Darwesh E Albogami R Taha A. Knowledge, attitude, and practice towards OTC drugs (OTC) use among adult population in Jeddah, Saudi Arabia. Middle East J Fam Med. (2022) 20. 10.5742/MEWFM.2022.9525056

[32] Watanabe K. Recent social background and consumer views on OTC drugs and self-medication. Yakugaku Zasshi J Pharm Soc Japan. (2020) 140:423–34. 10.1248/yakushi.19-00117 32115565

[33] Hui G Li Y. Investigation and analysis of residents' OTC drug purchase behavior. Chin Pharm. (2014) 25:314–7.

[34] Hong C Hu H. Survey on residents' perception of community pharmacy pharmacists providing pharmaceutical services when purchasing non prescription drugs in Yinchuan City. Chin Pharm. (2016) 27:4197–9.

[35] Jianzhou Y Ge Q Zhu Z Shao R. Research on OTC management system in Taiwan based on consumer guidance. Chin Pharm. (2019) 30:2161–5.

[36] Jiu W Zhang B Duan Y. Investigation on self medical behavior of urban residents. Med Philos A. (2015) 36:42–4.

[37] Zhuqing Z Shen Z Ding S Zheng F Duan Y Luo A. Research status of self medication in China based on bibliometrics. J Centr South Univ. (2017) 42:434–9. 10.11817/j.issn.1672-7347.2017.04.011 28490702

[38] Duong TV Aringazina A Baisunova G Nurjanah N Pham TV Pham KM. Development and validation of a new short-form health literacy instrument (HLS-SF12) for the general public in six Asian countries. Health Lit Res Pract. (2019) 3:e91–102. 10.3928/24748307-20190225-01 31294310 PMC6607763

[39] Duong TV Nguyen TTP Pham KM Nguyen KT Giap MH Tran TDX. Validation of the short-form health literacy questionnaire (HLS-SF12) and its determinants among people living in rural areas in Vietnam. Int J Environ Res Public Health. (2019) 16:3346. 10.3390/ijerph16183346 31514271 PMC6765800

[40] Cheong PL Wang H Cheong W Lam MI. Health literacy among filipino domestic workers in Macao. Healthcare. (2021) 9:1449. 10.3390/healthcare9111449 34828495 PMC8621908

[41] Zhang W Shen X Li T Li N Sun Y Zhu S. Intention to pay for vaccination and influencing factors of general residents: a national cross-sectional study. Int J Environ Res Public Health. (2022) 19:11154. 10.3390/ijerph191811154 36141428 PMC9517589

[42] HLS-EU Consortium Comparative Report of Health Literacy in Eight EU Member States. The European Health Literacy Project 2009–2012. HLS-EU Consortium Comparative Report of Health Literacy in Eight EU Member States (2012). Available online at: http://cpme.dyndns.org:591/adopted/2015/Comparative_report_on_health_literacy_in_eight_EU_member_states.pdf (accessed August 15, 2021).

[43] Mai J Yibo W Ling Z Lina L Xinying S. Health literacy and personality traits in two types of family structure-a cross-sectional study in China. Front Psychol. (2022) 13:835909. 10.3389/fpsyg.2022.835909 35548527 PMC9083055

[44] Rammstedt B John OP. Measuring personality in one minute or less: a 10-item short version of the Big Five inventory in English and German. J Res Pers. (2007) 41:203–12. 10.1016/j.jrp.2006.02.001

[45] Carciofo R Yang J Song N Du F Zhang K. Psychometric evaluation of Chinese-language 44-item and 10-item Big Five personality inventories, including correlations with chronotype, mindfulness and mind wandering. PLoS ONE. (2016) 11:e0149963. 10.1371/journal.pone.0149963 26918618 PMC4769279

[46] Nikčević AV Marino C Kolubinski DC Leach D Spada MM. Modelling the contribution of the Big Five personality traits, health anxiety, and COVID-19 psychological distress to generalised anxiety and depressive symptoms during the COVID-19 pandemic. J Affect Disord. (2021) 279:578–84. 10.1016/j.jad.2020.10.053 33152562 PMC7598311

[47] Airaksinen J Komulainen K Jokela M Gluschkoff K. Big Five personality traits and COVID-19 precautionary behaviors among older adults in Europe. Aging Health Res. (2021) 1:100038. 10.1016/j.ahr.2021.100038 34568860 PMC8450054

[48] McCrae RR Costa PT. Validation of the five-factor model of personality across instruments and observers. J Pers Soc Psychol. (1987) 52:81–90. 10.1037/0022-3514.52.1.81 3820081

[49] Heilmann K Hinrichs R Herke M Richter M Rathmann K. Die bedeutung der “Big Five”-persönlichkeitsmerkmale für die subjektive gesundheit und lebenszufriedenheit im jugendalter: ergebnisse des nationalen bildungspanels (NEPS) [The importance of the “Big Five” personality traits for subjective health and life satisfaction in adolescence: results of the national educational panel study (NEPS)]. Gesundheitswesen. (2021) 83:8–16. 10.1055/a-1068-2280 31923923

[50] Jia X Huang Y Yu W Ming WK Qi F Wu Y. A moderated mediation model of the relationship between family dynamics and sleep quality in college students: the role of Big Five personality and only-child status. Int J Environ Res Public Health. (2022) 19:3576. 10.3390/ijerph19063576 35329263 PMC8953608

[51] Jiang J Hong Y Zhang T Yang Z Lin T Liang Z. Comparing the measurement properties of the EQ-5D-5L and the EQ-5D-3L in hypertensive patients living in rural China. Qual Life Res. (2021) 30:2045–60. 10.1007/s11136-021-02786-5 33821418

[52] Hong JY Kim SY Chung KS Kim EY Jung JY Park MS. Factors associated with the quality of life of Korean COPD patients as measured by the EQ-5D. Qual Life Res. (2015) 24:2549–58. 10.1007/s11136-015-0979-6 25841978

[53] Cha AS Law EH Shaw JW Pickard AS. A comparison of self-rated health using EQ-5D VAS in the United States in 2002 and 2017. Qual Life Res. (2019) 28:3065–9. 10.1007/s11136-019-02249-y 31321671

[54] Yabin X Ma A. A study on the reliability and validity of the Chinese version of the European five dimensional health scale EQ-5D-5L. Shanghai Pharm. (2013) 34:40–3. 10.3969/j.issn.1006-1533.2013.09.021

[55] Li X Duan T Lai Y Wang X Yang L Su X. Status of medication literacy and its related factors among undergraduate students in Shanxi Province, China: a cross-sectional study. J Clin Pharm Ther. (2022) 47:1201–11. 10.1111/jcpt.13655 35347725

[56] Chen G Gully SM Eden D. Validation of a new general self-efficacy scale. Organ Res Methods. (2001) 4:62–83. 10.1177/109442810141004

[57] Xiao F Chen X. Study on the reliability and validity of the general self-efficacy scale (NGSES). J Mudanjiang Norm Coll. (2012) 2012:127–9. 10.13815/j.cnki.jmtc(pss).2012.04.042

[58] Tinaz S Elfil M Kamel S Aravala SS Louis ED Sinha R. Goal-directed behavior in individuals with mild Parkinson's disease: role of self-efficacy and self-regulation. Clin Park Relat Disord. (2020) 3:100051. 10.1016/j.prdoa.2020.100051 34113841 PMC8189568

[59] Schwarzer R Aristi B. Optimistic self-beliefs: assessment of general perceived self-efficacy in thirteen cultures. World Psychol. (1997) 3:177–90.

[60] Suhua Z. Effect of Family Nursing Intervention on Social Support, Self-Efficacy and Quality of Life of Low Vision Patients. Southern Medical University (2016).

[61] Ge P Liu ST Xu SX Zhang JZ Lai YJ Fu RC. The influence of parents on medication adherence of their children in China: a cross-sectional online investigation based on health belief model. Front Public Health. (2022) 10:845032. 10.3389/fpubh.2022.845032 35493366 PMC9046660

[62] Mei D Deng Y Li Q Lin Z Jiang H Zhang J. Current status and influencing factors of eating behavior in residents at the age of 18~60: a cross-sectional study in China. Nutrients. (2022) 14:2585. 10.3390/nu14132585 35807764 PMC9268282

[63] Arnastauskait J Ruzgas T Branas M. An Exhaustive Power Comparison of Normality Tests. (2021). 10.3390/math9070788

[64] Podsakoff PM MacKenzie SB Lee JY Podsakoff NP. Common method biases in behavioral research: a critical review of the literature and recommended remedies. J Appl Psychol. (2003) 88:879–903. 10.1037/0021-9010.88.5.879 14516251

[65] Ayalew MB. Self-medication practice in Ethiopia: a systematic review. Pat Prefer Adher. (2017) 11:401–13. 10.2147/PPA.S131496 28280312 PMC5338978

[66] Chang J Wang Q Fang Y. Socioeconomic differences in self-medication among middle-aged and older people: data from the China health and retirement longitudinal study. BMJ Open. (2017) 7:e017306. 10.1136/bmjopen-2017-017306 29259056 PMC5778336

[67] Abdi A Faraji A Dehghan F Khatony A. Prevalence of self-medication practice among health sciences students in Kermanshah, Iran. BMC Pharmacol Toxicol. (2018) 19:36. 10.1186/s40360-018-0231-4 29970167 PMC6029137

[68] Tabiei S. Self-medication with drug amongst university students of Birjand. Mod Care J. (2012) 9:371–8.

[69] Vahedi S Jalali FS Bayati M Delavari S. Predictors of self-medication in Iran: a notional survey study. Iran J Pharm Res. (2021) 20:348–58. 10.22037/ijpr.2020.113601.14394 34400964 PMC8170743

[70] Lei X Jiang H Liu C Ferrier A Mugavin J. Self-Medication practice and associated factors among residents in Wuhan, China. Int J Environ Res Public Health. (2018) 15:68. 10.3390/ijerph15010068 29300318 PMC5800167

[71] Zhe C Fan H Wang W Xiong W. Analysis of emergency management measures in large public hospitals during the high prevalence of novel coronavirus pneumonia. Chin Emerg Med. (2022) 42:604–9.

[72] Gelayee DA. Self-Medication pattern among social science university students in northwest Ethiopia. J Pharm. (2017) 2017:8680714. 10.1155/2017/8680714 28191360 PMC5278208

[73] Goh LY Vitry AI Semple SJ Esterman A Luszcz MA. Self-medication with over-the-counter drugs and complementary medications in South Australia's elderly population. BMC Complement Altern Med. (2009) 9:42. 10.1186/1472-6882-9-42 19906314 PMC2778637

[74] Al-Ghamdi S Alfauri TM Alharbi MA Alsaihati MM Alshaykh MM Alharbi AA. Current self-medication practices in the Kingdom of Saudi Arabia: an observational study. Pan Afr Med J. (2020) 37:51. 10.11604/pamj.2020.37.51.24098 33209178 PMC7648480

[75] Guangyu H Qiu W. From guidelines to practice: promoting risk communication and community participation in the prevention and control of COVID-19. Chin J Evid Based Med. (2020) 20:719–22. 32445287 10.1111/jebm.12387 PMC7280730

[76] Cybulski M Cybulski L Krajewska-Kulak E Orzechowska M Cwalina U. Preferences and attitudes of older adults of Bialystok, Poland toward the use of over-the-counter drugs. Clin Interv Aging. (2018) 13:623–32 10.2147/CIA.S158501 29692605 PMC5901153

[77] Martín-Pérez M Hernández Barrera V López de Andrés A Jiménez-Trujillo I Jiménez-García R Carrasco-Garrido P. Predictors of medication use in the Roma population in Spain: a population-based national study. Public Health. (2015) 129:453–9. 10.1016/j.puhe.2015.01.028 25795016

[78] Alzahrani M Alhindi T Almutairi A Aldajani M Sami W. Frequency of using non-prescribed medication in Majmaah city, Saudi Arabia—a cross sectional study. J Pak Med Assoc. (2015) 65:825–8. 10.7707/hmj.552 26228324

[79] Huan Y. Regional differences and dynamic evolution of the allocation level of quality medical resources in China. Northwest Popul. (2022) 43:92–103. 10.15884/j.cnki.issn.1007-0672.2022.04.008

[80] Jacob L Kostev K. Prevalence of pain medication prescriptions in France, Germany, and the UK—a cross-sectional study including 4,270,142 patients. Postgrad Med. (2018) 130:32–6. 10.1080/00325481.2018.1391658 29022417

[81] Lins S Aquino S. Development and initial psychometric properties of a panic buying scale during COVID-19 pandemic. Heliyon. (2020) 6:e04746. 10.1016/j.heliyon.2020.e04746 32895636 PMC7467094

[82] Nguyen TNM Laetsch DC Chen LJ Haefeli WE Meid AD Brenner H. Pain severity and analgesics use in the community-dwelling older population: a drug utilization study from Germany. Eur J Clin Pharmacol. (2020) 76:1695–707. 10.1007/s00228-020-02954-5 32648116 PMC7661425

[83] Antonov K Isacson D. Use of analgesics in Sweden–the importance of sociodemographic factors, physical fitness, health and health-related factors, and working conditions. Soc Sci Med. (1996) 42:1473–81. 10.1016/0277-9536(96)87321-7 8771630

[84] Sarganas G Buttery AK Zhuang W Wolf IK Grams D Rosario AS. Prevalence, trends, patterns and associations of analgesic use in Germany. BMC Pharmacol Toxicol. (2015) 16:28. 10.1186/s40360-015-0028-7 26428626 PMC4591581

[85] Dale O Borchgrevink PC Fredheim OM Mahic M Romundstad P Skurtveit S. Prevalence of use of non-prescription analgesics in the Norwegian HUNT3 population: impact of gender, age, exercise and prescription of opioids. BMC Public Health. (2015) 15:461. 10.1186/s12889-015-1774-6 25934132 PMC4428499

[86] Matin MA Khan WA Karim MM Ahmed S John-Langba J Sankoh OA. What influences antibiotic sales in rural Bangladesh? A drug dispensers' perspective. J Pharm Policy Pract. (2020) 13:20. 10.1186/s40545-020-00212-8 32514362 PMC7268404

[87] Taybeh E Al-Alami Z Alsous M Rizik M Alkhateeb Z. The awareness of the Jordanian population about OTC medications: a cross-sectional study. Pharmacol Res Perspect. (2019) 8:e00553. 10.1002/prp2.553 31890226 PMC6934420

[88] Rathish D Wickramasinghe ND. Prevalence, associated factors and reasons for antibiotic self-medication among dwellers in Anuradhapura: a community-based study. Int J Clin Pharm. (2020) 42:1139–44. 10.1007/s11096-020-01065-6 32458226

[89] Lescure D Paget J Schellevis F van Dijk L. Determinants of self-medication with antibiotics in European and Anglo-Saxon countries: a systematic review of the literature. Front Public Health. (2018) 6:370. 10.3389/fpubh.2018.00370 30619809 PMC6304439

[90] Ming Y Xing H Chen D Du Y. Problems and countermeasures of prescription drug management in social retail pharmacy. China Pharm. (2005) 8:462–4.

[91] Su F. Research on the Current Situation and Countermeasures of the Development of Clinical Pharmacy Service in the Third Class A Hospital of Jilin Province. Changchun University of Traditional Chinese Medicine (2018).

[92] Villako P Volmer D Raal A. Factors influencing purchase of and counselling about prescription and OTC medicines at community pharmacies in Tallinn, Estonia. Acta Pol Pharm. (2012) 69:335–40. 22568049

[93] Ramadan B. Knowledge and attitude of medical students toward self-medication. J Popul Ther Clin Pharmacol. (2022) 28:e83–91. 10.47750/jptcp.2022.862 35213107

[94] Pileggi C Mascaro V Bianco A Pavia M. Over-the-Counter drugs and complementary medications use among children in Southern Italy. Biomed Res Int. (2015) 2015:413912. 10.1155/2015/413912 26106606 PMC4464002

[95] Wensheng Z Jiang H Ai H Luo J Wang X. Regional differences in the supply level of basic medical and health resources in China. Geogr Res. (2015) 34:2049–60.

[96] Zhengqiang Q. Chronic disease management is the key direction for retail pharmacy development. Shanghai Pharm. (2017) 38:60–4. 10.3969/j.issn.1006-1533.2017.07.024

[97] Xiangyan X Shi H. Analysis of the current situation of medication safety awareness level and its related influencing factors among elderly patients with chronic diseases. Chin J Clin Phys. (2022) 50:589–91.

[98] Panpan L Zhang J Zhang G Xiu X Wang A. Self-management behavior of diabetic foot patients based on health ecology model. J Qingdao Univer. (2020) 56:678–82. 10.11712/jms.2096-5532.2020.56.168

[99] Linkievicz NM Sgnaolin V Engroff P Jardim BGG Neto CA. Association between Big Five personality factors and medication adherence in the elderly. Trends Psychiatry Psychother. (2022) 44:e20200143. 10.47626/2237-6089-2020-0143 33834686 PMC9907374

[100] Yuting L. A Study on the Influence of Big Five Personality Traits on Elementary School Teachers' Self-Efficacy. Shanghai Normal University (2022).

[101] Jie L Dai X. A preliminary development of the Chinese adjective Big Five personality scale I: theoretical framework and test reliability. Chin J Clin Psychol. (2015) 23:381–5. 10.16128/j.cnki.1005-3611.2015.03.001

[102] Kamran A Sharifirad G Shafaeei Y Azadbakht L. Sodium intake prediction with health promotion model constructs in rural hypertensive patients. Indian J Public Health. (2015) 59:102–8. 10.4103/0019-557X.157517 26021646

[103] Weimei S Ren H Zhang Z Su C Li F Qiu L. Analysis of health literacy and influencing factors of residents in Yangqu county, Taiyuan city. Mod Prev Med. (2020) 47:2025–9.

[104] Kai S. Research on health literacy level of college students and its influencing factors in the context of health China action. Sports Sci Technol. (2022) 43:116–8. 10.14038/j.cnki.tykj.2022.03.013

[105] Jiping H Yang X Wang X Zheng A Yang J Xu X. Medication health literacy of major caregivers of children in Chinese families: a national survey. Med Guide. (2021) 40:1287–95. 10.3870/j.issn.1004-0781.2021.09.024

[106] Mondesir FL Levitan EB Malla G Mukerji R Carson AP Safford MM. Patient perspectives on factors influencing medication adherence among people with coronary heart disease (CHD) and CHD risk factors. Pat Prefer Adher. (2019) 13:2017–27. 10.2147/PPA.S222176 31819383 PMC6890172

[107] Youqin C Ji X Zhao X Peng J Lu B Zheng C. Internal path analysis of college students' healthy lifestyle based on structural equation model: mediating effect of self-efficacy. Mod Prev Med. (2021) 48:2239–42.

[108] Kato T. [Role of pharmacists and student pharmacists in educating and providing advice about over the counter (OTC) medications]. Yakugaku Zasshi. (2014) 134:223–35. 10.1248/yakushi.13-00210 24492226

[109] Kim HJ Yang YM Choi EJ. Use patterns of over-the-counter (OTC) medications and perspectives on OTC medications among Korean adult patients with chronic diseases: gender and age differences. Pat Prefer Adher. (2018) 12:1597–606. 10.2147/PPA.S173873 30214162 PMC6118289

[110] Hongxia L Zhu Y. Talking about the use instructions of non prescription drugs. Digest World Latest Med Inf. (2015) 15:249. 10.3969/j.issn.1671-141.2015.76.224

