# Supplementary material for: Self-medication in Chinese residents and the related factors of whether or not they would take suggestions from medical staff as an important consideration during self-medication
Source: Front Public Health. 2022 Dec 22;10:1074559. doi: 10.3389/fpubh.2022.1074559 (PMC9814121; doi:10.3389/fpubh.2022.1074559)
Supplement: Supplementary file 1 [file Table_1.DOCX]

**Supplements**

Supplement 1 Variable assignment

| **Variable** | **Assignment** |
| --- | --- |
| Gender | 0= Male，1= Female |
| Location | 0= Eastern China，1= Midwest China |
| Residential area | 0= Rural，1= Urban |
| Age | 0=19-35，1=36-59，2= 60 or higher |
| Education | 0= under college degree，1= College degree or higher |
| Monthly income per capita | 0= ¥4500 or lower，1= ¥4501 or higher |
| Chronic disease or not | 0= No，1= Yes |
| NGSES | 0= Low score group，1= High score group |
| HLS-SF12 | 0= Low score group，1= High score group |
| EQ-5D-VAS | 0= Low score group，1= High score group |
| Extraversion | 0= Low score group，1= High score group |
| Agreeableness | 0= Low score group，1= High score group |
| Conscientiousness | 0= Low score group，1= High score group |
| Neuroticism | 0= Low score group，1= High score group |
| Openness | 0= Low score group，1= High score group |
| Medical personnel suggestions | 0= Not an important consideration，1= An important consideration |
| NSAIDs | 0= Not purchased and used oneself，1= Purchased and used oneself |
| Antibacterials | 0= Not purchased and used oneself，1= Purchased and used oneself |

Supplement 2 One-way binary logistic regression analysis results of Vitamins and Minerals drugs and social demography characteristics

| Type | Variable | β | *P* | OR | 95%CI*(Lower）* | 95%CI*(Upper）* |
| --- | --- | --- | --- | --- | --- | --- |
| Vitamins and Minerals | Gender（The control group is male） |  |  |  |  |  |
|  | Female | 0.267 | **＜0.001** | 1.305 | 1.203 | 1.417 |
|  | Location within three months（The control group is eastern China） |  |  |  |  |  |
|  | Midwest China | 0.060 | 0.148 | 1.062 | 0.979 | 1.152 |
|  | Chronic disease or not（The control group is No） |  |  |  |  |  |
|  | Yes | -0.161 | **0.002** | 0.851 | 0.769 | 0.942 |
|  | Residential area（The control group is rural） |  |  |  |  |  |
|  | Urban | 0.417 | **＜0.001** | 1.517 | 1.384 | 1.662 |
|  | Self assessment of health（The control group is low score group） |  |  |  |  |  |
|  | High score group | 0.097 | **0.021** | 1.102 | 1.015 | 1.198 |
|  | Monthly income per capita（The control group is ¥4500 or lower） |  |  |  |  |  |
|  | ≥¥4501 | 0.248 | **＜0.001** | 1.281 | 1.180 | 1.390 |
|  | Age range（The control group is 19-35 years old） |  |  |  |  |  |
|  | 36-59 | -0.145 | **0.001** | 0.865 | 0.793 | 0.944 |
|  | ≥60 | -0.486 | **＜0.001** | 0.615 | 0.537 | 0.704 |
|  | Education（The control group is under college degree） |  |  |  |  |  |
|  | College degree or higher | 0.421 | **＜0.001** | 1.523 | 1.401 | 1.656 |

Supplement 3 One-way binary logistic regression analysis results of external drug for skin and social demography characteristics

| Type | Variable | β | *P* | OR | 95%CI*(Lower）* | 95%CI*(Upper）* |
| --- | --- | --- | --- | --- | --- | --- |
| External Drug for Skin | Gender（The control group is male） |  |  |  |  |  |
|  | Female | 0.014 | 0.745 | 1.014 | 0.932 | 1.103 |
|  | Location within three months（The control group is eastern China） |  |  |  |  |  |
|  | Midwest China | 0.080 | 0.063 | 1.083 | 0.996 | 1.178 |
|  | Chronic disease or not（The control group is No） |  |  |  |  |  |
|  | Yes | 0.205 | **＜0.001** | 1.227 | 1.107 | 1.360 |
|  | Residential area（The control group is rural） |  |  |  |  |  |
|  | Urban | 0.045 | 0.344 | 1.046 | 0.952 | 1.150 |
|  | Self assessment of health（The control group is low score group） |  |  |  |  |  |
|  | High score group | 0.025 | 0.565 | 1.025 | 0.941 | 1.117 |
|  | Monthly income per capita（The control group is ¥4500 or lower） |  |  |  |  |  |
|  | ≥¥4501 | -0.046 | 0.288 | 0.955 | 0.878 | 1.039 |
|  | Age range（The control group is 19-35 years old） |  |  |  |  |  |
|  | 36-59 | 0.163 | **＜0.001** | 1.177 | 1.076 | 1.287 |
|  | ≥60 | 0.029 | 0.685 | 1.029 | 0.896 | 1.183 |
|  | Education（The control group is under college degree） |  |  |  |  |  |
|  | College degree or higher | -0.018 | 0.676 | 0.982 | 0.901 | 1.070 |

Supplement 4 One-way binary logistic regression analysis results of digestive system medication and social demography characteristics

| Type | Variable | β | *P* | OR | 95%CI*(Lower）* | 95%CI*(Upper）* |
| --- | --- | --- | --- | --- | --- | --- |
| Digestive System Medication | Gender（The control group is male） |  |  |  |  |  |
|  | Female | -0.131 | 0.003 | 0.878 | 0.806 | 0.956 |
|  | Location within three months（The control group is eastern China） |  |  |  |  |  |
|  | Midwest China | -0.017 | 0.693 | 0.983 | 0.903 | 1.070 |
|  | Chronic disease or not（The control group is No） |  |  |  |  |  |
|  | Yes | 0.374 | **＜0.001** | 1.453 | 1.311 | 1.611 |
|  | Residential area（The control group is rural） |  |  |  |  |  |
|  | Urban | -0.025 | 0.610 | 0.976 | 0.887 | 1.073 |
|  | Self assessment of health（The control group is low score group） |  |  |  |  |  |
|  | High score group | -0.136 | 0.002 | 0.873 | 0.800 | 0.951 |
|  | Monthly income per capita（The control group is ¥4500 or lower） |  |  |  |  |  |
|  | ≥¥4501 | 0.076 | 0.078 | 1.079 | 0.991 | 1.175 |
|  | Age range（The control group is 19-35 years old） |  |  |  |  |  |
|  | 36-59 | 0.178 | **＜0.001** | 1.195 | 1.091 | 1.309 |
|  | ≥60 | 0.434 | **＜0.001** | 1.543 | 1.346 | 1.770 |
|  | Education（The control group is under college degree） |  |  |  |  |  |
|  | College degree or higher | -0.164 | **＜0.001** | 0.849 | 0.778 | 0.926 |

Supplement 5 One-way binary logistic regression analysis results of Chinese patent medicine and social demography characteristics

| Type | Variable | β | *P* | OR | 95%CI*(Lower）* | 95%CI*(Upper）* |
| --- | --- | --- | --- | --- | --- | --- |
| Chinese Patent Medicine | Gender（The control group is male） |  |  |  |  |  |
|  | Female | 0.210 | **＜0.001** | 1.234 | 1.124 | 1.355 |
|  | Location within three months（The control group is eastern China） |  |  |  |  |  |
|  | Midwest China | -0.008 | 0.859 | 0.992 | 0.904 | 1.088 |
|  | Chronic disease or not（The control group is No） |  |  |  |  |  |
|  | Yes | 0.437 | **＜0.001** | 1.548 | 1.388 | 1.726 |
|  | Residential area（The control group is rural） |  |  |  |  |  |
|  | Urban | 0.329 | **＜0.001** | 1.390 | 1.248 | 1.547 |
|  | Self assessment of health（The control group is low score group） |  |  |  |  |  |
|  | High score group | -0.113 | **0.018** | 0.893 | 0.813 | 0.981 |
|  | Monthly income per capita（The control group is ¥4500 or lower） |  |  |  |  |  |
|  | ≥¥4501 | 0.019 | 0.683 | 1.019 | 0.929 | 1.118 |
|  | Age range（The control group is 19-35 years old） |  |  |  |  |  |
|  | 36-59 | 0.339 | **＜0.001** | 1.403 | 1.270 | 1.550 |
|  | ≥60 | 0.410 | **＜0.001** | 1.508 | 1.300 | 1.749 |
|  | Education（The control group is under college degree） |  |  |  |  |  |
|  | College degree or higher | -0.073 | 0.128 | 0.929 | 0.846 | 1.021 |

Supplement 6 One-way binary logistic regression analysis results of respiratory system medication and social demography characteristics

| Type | Variable | β | *P* | OR | 95%CI*(Lower）* | 95%CI*(Upper）* |
| --- | --- | --- | --- | --- | --- | --- |
| Respiratory System Medication | Gender（The control group is male） |  |  |  |  |  |
|  | Female | -0.219 | **＜0.001** | 0.804 | 0.722 | 0.894 |
|  | Location within three months（The control group is eastern China） |  |  |  |  |  |
|  | Midwest China | 0.027 | 0.615 | 1.028 | 0.924 | 1.143 |
|  | Chronic disease or not（The control group is No） |  |  |  |  |  |
|  | Yes | 0.444 | **＜0.001** | 1.559 | 1.379 | 1.763 |
|  | Residential area（The control group is rural） |  |  |  |  |  |
|  | Urban | -0.007 | 0.903 | 0.993 | 0.882 | 1.118 |
|  | Self assessment of health（The control group is low score group） |  |  |  |  |  |
|  | High score group | -0.234 | **＜0.001** | 0.791 | 0.711 | 0.880 |
|  | Monthly income per capita（The control group is ¥4500 or lower） |  |  |  |  |  |
|  | ≥¥4501 | 0.224 | **＜0.001** | 1.276 | 1.147 | 1.420 |
|  | Age range（The control group is 19-35 years old） |  |  |  |  |  |
|  | 36-59 | 0.207 | **＜0.001** | 1.230 | 1.095 | 1.382 |
|  | ≥60 | 0.670 | **＜0.001** | 1.954 | 1.666 | 2.293 |
|  | Education（The control group is under college degree） |  |  |  |  |  |
|  | College degree or higher | -0.136 | **0.014** | 0.873 | 0.784 | 0.972 |

Supplement 7 One-way binary logistic regression analysis results of antiallergic medication and social demography characteristics

| Type | Variable | β | *P* | OR | 95%CI*(Lower）* | 95%CI*(Upper）* |
| --- | --- | --- | --- | --- | --- | --- |
| Antiallergic Medication | Gender（The control group is male） |  |  |  |  |  |
|  | Female | 0.253 | **＜0.001** | 1.288 | 1.144 | 1.449 |
|  | Location within three months（The control group is eastern China） |  |  |  |  |  |
|  | Midwest China | -0.183 | **0.002** | 0.833 | 0.741 | 0.936 |
|  | Chronic disease or not（The control group is No） |  |  |  |  |  |
|  | Yes | 0.207 | **0.003** | 1.230 | 1.071 | 1.413 |
|  | Residential area（The control group is rural） |  |  |  |  |  |
|  | Urban | 0.280 | **＜0.001** | 1.323 | 1.154 | 1.516 |
|  | Self assessment of health（The control group is low score group） |  |  |  |  |  |
|  | High score group | 0.008 | 0.896 | 1.008 | 0.895 | 1.135 |
|  | Monthly income per capita（The control group is ¥4500 or lower） |  |  |  |  |  |
|  | ≥¥4501 | 0.132 | **0.027** | 1.141 | 1.015 | 1.282 |
|  | Age range（The control group is 19-35 years old） |  |  |  |  |  |
|  | 36-59 | 0.160 | **0.011** | 1.174 | 1.038 | 1.327 |
|  | ≥60 | -0.078 | 0.443 | 0.925 | 0.757 | 1.130 |
|  | Education（The control group is under college degree） |  |  |  |  |  |
|  | College degree or higher | 0.269 | **＜0.001** | 1.308 | 1.158 | 1.478 |

Supplement 8 One-way binary logistic regression analysis results of gynecological medication and social demography characteristics

| Type | Variable | β | *P* | OR | 95%CI*(Lower）* | 95%CI*(Upper）* |
| --- | --- | --- | --- | --- | --- | --- |
| Gynecological Medication | Location within three months（The control group is eastern China） |  |  |  |  |  |
|  | Midwest China | 0.080 | 0.063 | 1.083 | 0.996 | 1.178 |
|  | Chronic disease or not（The control group is No） |  |  |  |  |  |
|  | Yes | 0.297 | **0.001** | 1.345 | 1.125 | 1.609 |
|  | Residential area（The control group is rural） |  |  |  |  |  |
|  | Urban | 0.297 | **0.001** | 1.346 | 1.137 | 1.593 |
|  | Self assessment of health（The control group is low score group） |  |  |  |  |  |
|  | High score group | -0.172 | **0.021** | 0.842 | 0.727 | 0.974 |
|  | Monthly income per capita（The control group is ¥4500 or lower） |  |  |  |  |  |
|  | ≥¥4501 | 0.011 | 0.884 | 1.011 | 0.874 | 1.169 |
|  | Age range（The control group is 19-35 years old） |  |  |  |  |  |
|  | 36-59 | 0.761 | **＜0.001** | 2.140 | 1.835 | 2.496 |
|  | ≥60 | -0.177 | 0.234 | 0.838 | 0.626 | 1.121 |
|  | Education（The control group is under college degree） |  |  |  |  |  |
|  | College degree or higher | -0.363 | **＜0.001** | 0.695 | 0.601 | 0.805 |

Supplement 9 Logistic regression results of male residents

| Variable | β | SE | *Wald χ²* | *P* | OR | 95%CI (Lower） | 95%CI (Upper) |
| --- | --- | --- | --- | --- | --- | --- | --- |
| Age range（The control group is 19-35 years old） |  |  |  |  |  |  |  |
| ≥36 | 0.336 | 0.089 | 14.352 | **＜0.001** | 1.400 | 1.176 | 1.666 |
| Agreeableness（The control group is low score group） |  |  |  |  |  |  |  |
| High score group | 0.456 | 0.093 | 23.853 | **＜0.001** | 1.577 | 1.314 | 1.894 |
| Conscientiousness（The control group is low score group） |  |  |  |  |  |  |  |
| High score group | 0.286 | 0.096 | 8.948 | **0.003** | 1.331 | 1.104 | 1.605 |
| Health literacy（The control group is low in health literacy） |  |  |  |  |  |  |  |
| High | 0.289 | 0.094 | 9.548 | **0.002** | 1.335 | 1.112 | 1.604 |
| NGSES score（The control group is low score group） |  |  |  |  |  |  |  |
| High score group | 0.244 | 0.098 | 6.182 | **0.013** | 1.276 | 1.053 | 1.547 |

Supplement 10 Logistic regression results of female residents

| Variable | β | SE | *Wald χ²* | *P* | OR | 95%CI (Lower） | 95%CI (Upper) |
| --- | --- | --- | --- | --- | --- | --- | --- |
| Agreeableness（The control group is low score group） |  |  |  |  |  |  |  |
| High score group | 0.616 | 0.090 | 46.601 | **＜0.001** | 1.852 | 1.552 | 2.211 |
| Conscientiousness（The control group is low score group） |  |  |  |  |  |  |  |
| High score group | 0.342 | 0.092 | 13.786 | **＜0.001** | 1.408 | 1.175 | 1.687 |
| Health literacy（The control group is low in health literacy） |  |  |  |  |  |  |  |
| High | 0.220 | 0.092 | 5.728 | **0.017** | 1.246 | 1.041 | 1.492 |
| EQ-5D-VAS score（The control group is low score group） |  |  |  |  |  |  |  |
| High score group | 0.181 | 0.090 | 4.095 | **0.043** | 1.199 | 1.006 | 1.429 |
| NGSES score（The control group is low score group） |  |  |  |  |  |  |  |
| High score group | 0.318 | 0.097 | 10.678 | **0.001** | 1.375 | 1.136 | 1.664 |

Supplement 11 Logistic Regression Results of Residents Aged 35 or Lower

| Variable | β | SE | *Wald χ²* | *P* | OR | 95%CI (Lower） | 95%CI (Upper) |
| --- | --- | --- | --- | --- | --- | --- | --- |
| Gender（The control group is male） |  |  |  |  |  |  |  |
| Female | 0.346 | 0.089 | 15.228 | **＜0.001** | 1.413 | 1.188 | 1.681 |
| Agreeableness（The control group is low score group） |  |  |  |  |  |  |  |
| High score group | 0.583 | 0.093 | 39.289 | **＜0.001** | 1.792 | 1.493 | 2.150 |
| Openness（The control group is low score group） |  |  |  |  |  |  |  |
| High score group | 0.344 | 0.095 | 13.144 | **＜0.001** | 1.411 | 1.171 | 1.699 |
| Health literacy（The control group is low in health literacy） |  |  |  |  |  |  |  |
| High | 0.283 | 0.096 | 8.620 | **0.003** | 1.327 | 1.099 | 1.602 |
| NGSES score（The control group is low score group） |  |  |  |  |  |  |  |
| High score group | 0.193 | 0.097 | 3.940 | **0.047** | 1.212 | 1.002 | 1.466 |

Supplement 12 Logistic regression results of residents over 35 years old

| Variable | β | SE | *Wald χ²* | *P* | OR | 95%CI (Lower） | 95%CI (Upper) |
| --- | --- | --- | --- | --- | --- | --- | --- |
| Location within three months（The control group is eastern China） |  |  |  |  |  |  |  |
| Midwest China | 0.205 | 0.086 | 5.683 | **0.017** | 1.228 | 1.037 | 1.453 |
| Chronic disease or not（The control group is No） |  |  |  |  |  |  |  |
| Yes | 0.227 | 0.096 | 5.623 | **0.018** | 1.255 | 1.040 | 1.514 |
| Agreeableness（The control group is low score group） |  |  |  |  |  |  |  |
| High score group | 0.383 | 0.093 | 17.088 | **＜0.001** | 1.467 | 1.223 | 1.759 |
| Conscientiousness（The control group is low score group） |  |  |  |  |  |  |  |
| High score group | 0.524 | 0.093 | 31.917 | **＜0.001** | 1.688 | 1.408 | 2.025 |
| Health literacy（The control group is low in health literacy） |  |  |  |  |  |  |  |
| High | 0.243 | 0.092 | 6.901 | **0.009** | 1.275 | 1.064 | 1.528 |
| EQ-5D-VAS score（The control group is low score group） |  |  |  |  |  |  |  |
| High score group | 0.198 | 0.091 | 4.704 | **0.030** | 1.218 | 1.019 | 1.457 |
| NGSES score（The control group is low score group） |  |  |  |  |  |  |  |
| High score group | 0.367 | 0.097 | 14.203 | **＜0.001** | 1.443 | 1.192 | 1.746 |

Supplement 13 Logistic Regression Results of Rural Residents

| Variable | β | SE | *Wald χ²* | *P* | OR | 95%CI (Lower） | 95%CI (Upper) |
| --- | --- | --- | --- | --- | --- | --- | --- |
| Agreeableness（The control group is low score group） |  |  |  |  |  |  |  |
| High score group | 0.733 | 0.121 | 36.477 | **＜0.001** | 2.082 | 1.641 | 2.641 |
| Conscientiousness（The control group is low score group） |  |  |  |  |  |  |  |
| High score group | 0.468 | 0.123 | 14.483 | **＜0.001** | 1.597 | 1.255 | 2.033 |
| NGSES score（The control group is low score group） |  |  |  |  |  |  |  |
| High score group | 0.296 | 0.125 | 5.579 | **0.018** | 1.344 | 1.052 | 1.718 |

Supplement 14 Logistic Regression Results of Urban Residents

| Variable | β | SE | Wald χ² | *P* | OR | 95%CI (Lower） | 95%CI (Upper) |
| --- | --- | --- | --- | --- | --- | --- | --- |
| Gender（The control group is male） |  |  |  |  |  |  |  |
| Female | 0.222 | 0.073 | 9.218 | **0.002** | 1.249 | 1.082 | 1.441 |
| Chronic disease or not（The control group is No） |  |  |  |  |  |  |  |
| Yes | 0.362 | 0.100 | 13.065 | **＜0.001** | 1.436 | 1.180 | 1.747 |
| Extraversion（The control group is low score group） |  |  |  |  |  |  |  |
| High score group | 0.234 | 0.082 | 8.097 | **0.004** | 1.263 | 1.075 | 1.484 |
| Agreeableness（The control group is low score group） |  |  |  |  |  |  |  |
| High score group | 0.440 | 0.077 | 32.529 | **＜0.001** | 1.553 | 1.335 | 1.806 |
| Conscientiousness（The control group is low score group） |  |  |  |  |  |  |  |
| High score group | 0.199 | 0.080 | 6.181 | **0.013** | 1.220 | 1.043 | 1.427 |
| Health literacy（The control group is low in health literacy） |  |  |  |  |  |  |  |
| High | 0.303 | 0.079 | 14.591 | **＜0.001** | 1.355 | 1.159 | 1.583 |
| EQ-5D-VAS score（The control group is low score group） |  |  |  |  |  |  |  |
| High score group | 0.274 | 0.074 | 12.956 | **＜0.001** | 1.315 | 1.333 | 1.527 |
| NGSES score（The control group is low score group） |  |  |  |  |  |  |  |
| High score group | 0.257 | 0.081 | 9.935 | **0.002** | 1.249 | 1.082 | 1.441 |
